# Environmental Interactions and Epistasis Are Revealed in the Proteomic Responses to Complex Stimuli

**DOI:** 10.1371/journal.pone.0134099

**Published:** 2015-08-06

**Authors:** Parimal Samir, Rahul, James C. Slaughter, Andrew J. Link

**Affiliations:** 1 Department of Biochemistry, Vanderbilt University School of Medicine, Nashville, Tennessee, United States of America; 2 Department of Applied Mathematics, University of Waterloo, Waterloo, Ontario, Canada; 3 Department of Biostatistics, Vanderbilt University School of Medicine, Nashville, Tennessee, United States of America; 4 Department of Pathology, Microbiology and Immunology, Vanderbilt University School of Medicine, Nashville, Tennessee, United States of America; 5 Department of Chemistry, Vanderbilt University, Nashville, Tennessee, United States of America; CRG, SPAIN

## Abstract

Ultimately, the genotype of a cell and its interaction with the environment determine the cell’s biochemical state. While the cell’s response to a single stimulus has been studied extensively, a conceptual framework to model the effect of multiple environmental stimuli applied concurrently is not as well developed. In this study, we developed the concepts of environmental interactions and epistasis to explain the responses of the *S*. *cerevisiae* proteome to simultaneous environmental stimuli. We hypothesize that, as an abstraction, environmental stimuli can be treated as analogous to genetic elements. This would allow modeling of the effects of multiple stimuli using the concepts and tools developed for studying gene interactions. Mirroring gene interactions, our results show that environmental interactions play a critical role in determining the state of the proteome. We show that individual and complex environmental stimuli behave similarly to genetic elements in regulating the cellular responses to stimuli, including the phenomena of dominance and suppression. Interestingly, we observed that the effect of a stimulus on a protein is dominant over other stimuli if the response to the stimulus involves the protein. Using publicly available transcriptomic data, we find that environmental interactions and epistasis regulate transcriptomic responses as well.

## Introduction

In their native environments, cells continuously respond to a complexity of environmental stimuli. These include ambient temperature fluctuations, nutrient availability, signaling molecules, and physical forces. In response, cells adjust their biochemical state through multiple mechanisms including the differential production, modification, and degradation of transcripts and proteins [1,2,3,4,5]. Both extracellular signaling and the metabolic environment strongly influence a cell’s growth and responses to therapeutic treatments [6,7,8,9]. Model organisms have been used extensively to study cellular responses to individual and combinations of environmental stimuli [1,10,11,12,13,14,15,16,17]. We extend these approaches by developing and testing a novel conceptual framework to study proteomic responses of cells to the combinatorial effects of multiple concurrent environmental factors. We have modeled our analysis of these complex environmental interactions using the concepts of gene interaction and genetic epistasis.

Gene interaction is defined as the interaction between genes at different loci that affect the same characteristic or a trait [18]. Classically, genetic epistasis is referred to a type of gene interaction in which a mutation at one locus masks or suppresses the phenotype of a mutation at a different locus [18,19]. To test the independence of the effects of individual genes, genetic epistasis has also been defined mathematically as a type of gene interaction in which the combined effect of two or more mutations is not the sum of the effects of the individual mutations [20,21,22].

Conceptually, the problem of studying multiple concurrent environmental stimuli is similar to the problem of studying the effects of multiple genetic mutations. The product of a gene functions as part of one or more functional modules in concert with the products of many genes. The changes in a gene, for example its loss of function or gain of function, affects the phenotype due to the changes in the activity of the functional modules. If multiple genetic alterations are present, the total effect is due to the integration of the effects of the individual alterations through the functional modules. Similarly, environmental stimuli affect the biochemical state of the cells through specific sensing, signaling, and response modules. Concurrent application of multiple environmental stimuli, similar to the genetic alterations, requires the integration of information from these modules to mount an optimal response. By considering an environmental stimulus as an analogue of a gene, we hypothesized that the concepts of gene interaction and epistasis can be extrapolated to devise a conceptual framework for studying the combined effects of multiple concurrent stimuli. There are several benefits of using this approach; (1) all the genetic, biochemical, and computational tools and concepts developed for studying gene interactions would become available for studying the effects of the environment, (2) it would allow for easier mechanistic interpretation of the responses to complex environmental stimuli, (3) the contributions of an individual stimulus to altering biological processes can be more easily elucidated, and (4) it would provide a unifying framework for studying gene-gene, gene-environment and environment-environment interactions.

In this study, we define an environmental interaction as the interaction between different environmental stimuli that affect the same observable characteristic or trait. Similar to the statistical definition of genetic epistasis, environmental epistasis is an environmental interaction in which the effects of the individual stimuli are not independent of each other [20,21,22]. To test our hypothesis, we used the yeast *S*. *cerevisiae* and grew cells at standard conditions (glucose, 30°C) and changed growth conditions to either high temperature (37°C, HT stimulus) or the non-fermentable carbon source glycerol (G stimulus), and concurrently with both environmental stimuli (glycerol, 37°C, HT+G stimuli) (S1 Fig). Using precise quantitative proteomics of the *S*. *cerevisiae* proteome and the changes in protein abundance as the readouts of the interactions, we show that environmental interactions and epistasis play central roles in determining the state of the proteome in response to multiple, concurrent environmental stimuli. We also show that, using the dominance of one stimulus over another, environmental interactions can be used to identify proteins that are important for responding to a dominant stimulus. We validated our approach using an independent publicly available transcriptomic dataset.

## Experimental Procedures

### Strains and Media

All experiments used the diploid *S*. *cerevisiae* strain BY4743, which has been previously described [23]. Cells were grown using standard techniques [24].

### Growth rate analysis

Cells were grown in 96 well plates in 100 μL cultures (10 μL of starter culture and 90 μL of fresh media) with continuous shaking in a BioTek Synergy 4 Hybrid Microplate Reader for 10 h. Growth rates were assayed in 8 conditions: (1) Synthetic complete medium with glucose (ScD) at 30°C, (2) ScD at 37°C, (3) Synthetic complete medium with glycerol (ScG) at 30°C, (4) ScG at 37°C, (5) Yeast extract, peptone medium with glucose (YPD) at 30°C, (6) YPD at 37°C, (7) Yeast extract, peptone medium with glycerol (YPG) at 30°C, and (8) YPG at 37°C. Absorbance was measured at 660nm at 3 min intervals. Using custom *R* scripts, the doubling times were calculated from the linear regression curve through the log growth phase using the log of the absorbance and time of growth. A two-tailed *t*-test of independence with Bonferroni correction for the 11 comparisons (7 comparisons of the control, YPD at 30°C, to the test conditions, 3 comparisons of the observed concurrent double stimuli effect to the expected sum of individual stimulus effects, and 1 comparison of the observed concurrent three stimuli effect to the expected sum of the effects of the three individual stimulus) was used to calculate the statistical significance of a stimulus effect on the growth rate [25].

### Preparation of yeast protein extracts

Five mL of YPD (1% yeast extract, 2% peptone, 2% glucose) was inoculated with a single yeast colony from a YPD agar plate and grown overnight. Three replicates were grown under each growth condition: YPD at 30°C and 37°C and YPG at 30°C and 37°C. Fifty mL of YPD was inoculated with 50 μL of the overnight culture and grown at 30°C and 37°C. One hundred mL of YPG (1% yeast extract, 2% peptone, and 3% glycerol v/v) was inoculated with overnight cultures and grown at 30°C and 37°C. The cultures were grown with constant shaking at 175 rpm in Innova 44 shaker incubators (New Brunswick Scientific). For all four growth conditions, cells were harvested at mid-log phase as determined by OD_600_ measurements. Cells grown in YPD were harvested after 14 h, while cells grown in YPG were harvested after 24 h. All cultures were centrifuged at 2000 rpm for 5 min at 4°C using a Sorvall HLR6/H600A/HBB6 rotor in Sorvall RC-3B centrifuge and washed with ice cold deionized H_2_0. The cell pellets were resuspended in 1 mL ice cold wash buffer (10 mM Tris pH 8.0, 5 mM beta-mercaptoethanol, 500 mM ammonium chloride, 100 mM magnesium acetate) and lysed at 4°C using glass beads and a Bead Beater (BioSpec, Inc) for 10 min as previously described [26]. The whole cell extracts (WCE) were clarified by centrifugation at 20,000g for 15 min at 4°C, and a 200 μL aliquot of the cleared WCE was stored at -80°C.

### Isobaric tag for relative and absolute quantitation (iTRAQ) labeling

The total protein concentration was determined using a Bradford assay according to the manufacturer’s protocol (Sigma Aldrich). For each growth condition, 50 μg of total protein was mixed with 50 ng of bovine serum albumin (Thermo Scientific) as an internal standard. Each protein sample was acetone precipitated and resolublized in 25 μl iTRAQ dissolution buffer (500 mM triethylammonium bicarbonate, 0.1% sodium dodecyl sulfate). The proteins were reduced with tris(2-carboxyethyl)phosphine at 60°C for 60 min and the cysteines were derivatized with methyl methanethiosulfonate at room temperature for 10 min. All samples were digested with sequencing-grade modified trypsin (1:50; Promega Corporation) overnight at 37°C. Equal fractions of the tryptic digests from the three replicates grown in YPD at 30°C were pooled separately and used as a control for the iTRAQ experiments. Fifty μg of the pooled control and 50 μg of each of the replicates were used for iTRAQ labeling. The iTRAQ labeling reagents were resolublized in 150 μL anhydrous ethanol (Sigma Aldrich). 75 μL of iTRAQ reagent solutions were added to each 50 μg sample, incubated with shaking for 1 h at room temperature on an Eppendorf Thermomixer R, pooled, frozen, lyophilized, resolublized in 200 μL of buffer A (0.1% formic acid), and stored at -80°C.

### Liquid chromatography and mass spectrometry

The iTRAQ-labeled samples were analyzed with MudPIT as previously described [27]. Precursor ions were analyzed in the Orbitrap mass analyzer followed by four CID fragment ion scans in the ion trap and four HCD fragment ion scans (normalized collision energy = 45%) in the Orbitrap.

iTRAQ data analysis: RAW files generated by the MudPIT experiments were searched using the Sequest HT database search engine running under Proteome Discoverer v1.4 (Thermo Scientific) against a forward and reverse yeast protein database (S.cererevisiae_orf_trans_all_SGD.fasta.6718) with appended common contaminant sequences [28,29]. Beta-methylthiolation and iTRAQ modifications were included as constant modifications. Oxidation of methionine and tryptophan, and deamidation of glutamine and asparagine were used as variable modifications. Precursor mass tolerance was set to 3 Da and fragment mass tolerance was set to 0.8 Da. Protein assembly, reporter ion quantitation, and protein fold change calculations were done using ProteoIQ at 5% peptide and protein FDR (Premier Biosoft). Hierarchical clustering analysis was done using Cluster 3.0 [30]. Heatmaps were generated using Java Treeview [31]. Circos plots were generated as described in Krzywinski *et*. *al*. to visualize the genomic locations of the quantitated proteins [32]. For better visualization, only those regions of the genome that were quantitated in this study are shown. The mass spectrometry proteomics data have been deposited to the ProteomeXchange Consortium via the PRIDE partner repository with the dataset identifier PXD002371 [33].

### Environmental interaction analysis

All analysis was performed using *R* scripts to parse the fold change expression data to identify proteins that show specific expression patterns in response to complex environmental stimuli. For each protein, we used linear regression to test for any association of high temperature or glycerol using a model that included main effects for glycerol and temperature and the glycerol by temperature interaction. We used the effect size estimates and ANOVA *p-values* (3 degrees of freedom) calculated by the *lm* function and adjusted the *p-values* for a 5% FDR using the Benjamini-Hochberg procedure for finding differentially expressed proteins [34]. We used the adjusted *p-value* cut-off of 0.05 to determine statistical significance. If the overall adjusted *p-value* was greater than 0.05, we classified the proteins as non-responders. The positive and negative signs of the effect size estimates correspond to upregulation and downregulation, respectively, showing the direction of change. The remaining proteins were further classified into environmental interaction classes based upon the effect size estimate *p-values* and the direction of change. If the *p-value* of an estimate was less than 0.05, the protein was considered differentially expressed in response to that environmental stimulus.

To test if a protein is affected by environmental epistasis, the effect size estimates for the individual high temperature (HT) and glycerol stimuli (G) were summed, the combined standard error calculated as the square root of the sum of the squared standard errors, and a two-sample *t*-test of independence was used to compare the summed effect size estimate to the effect size estimate for the concurrent high temperature and glycerol stimuli (HT+G). If a *t*-test *p-value* was less than 0.05, the protein was assumed to be affected by environmental epistasis.

### Environmental interaction analysis of transcriptomic dataset

Normalized expression data described in Knijnenburg *et*. *al*. was downloaded [13]. The transcriptomic data were generated using haploid *S*. *cerevisiae* (CEN.PK113-7D MATa) cells grown in chemostat cultures [13]. We chose 4 culture conditions similar to our experimental design for further analysis. The culture conditions tested were: 1) with ammonium sulfate as the nitrogen source (n = 5), 2) with methionine as the nitrogen source (n = 3), 3) anaerobic conditions (n = 4), and 4) with methionine as the nitrogen source and anaerobic conditions concurrently (n = 3). Transcriptomic data from the cells grown with ammonium sulfate as the nitrogen source were used as the baseline control. The fold change was calculated by subtracting the average normalized expression data of baseline samples from the individual expression data. Finally, the genes were classified into various types of environmental interactions as described above.

### Co-expression network analysis

Sparse PArtial Correlation Estimation (SPACE) was used to build protein co-expression networks and identify the hub genes [35]. To account for outliers, the data were normalized using probabilistic quotient normalization and scaled using a generalized logarithmic scaling factor [36,37]. The data were scaled and centered to have a standard deviation of 1 and mean of 0 to remove any bias in the correlation analysis [38]. We estimated the partial correlation matrix using the space.dew method implemented in the SPACE *R* package [35]. We selected the default value of the tuning parameter for constructing the initial network [35]. The network was visualized in Cytoscape 3.1.1 [39].

## Results

**Fig 1:**
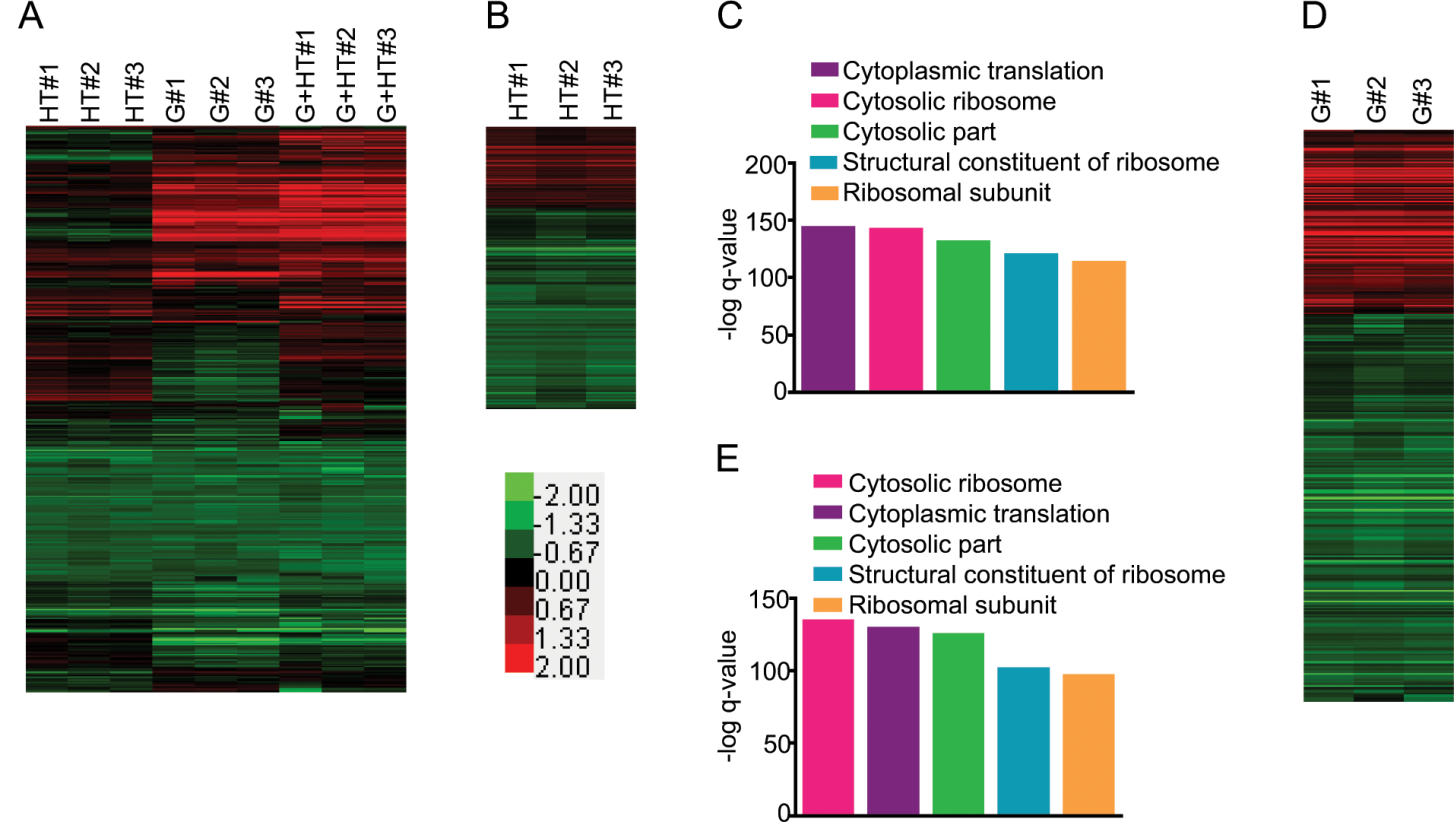
Proteomic responses to complex environmental stimuli. Diploid *S*. *cerevisiae* (BY4743) cells were grown in rich media under 4 conditions: 1) glucose as the carbon source at 30°C, 2) glycerol as the carbon source at 30°C, 3) glucose at 37°C, and 4) glycerol at 37°C. Three biological replicates for each growth conditions were performed. Fold changes were calculated from iTRAQ reporter ion intensities using reporter ion intensities from the pooled replicates of growth in glucose as the carbon source at 30°C as the baseline. The fold changes were log_2_ transformed for downstream analysis. The color bar shows the fold change range. A) Complete filtered proteomic dataset for high temperature stimulus (HT), glycerol stimulus (G), and concurrent glycerol and high temperature stimuli (HT+G) (Red: Up, Green: Down, Black: No change). The heatmap represents the fold changes of 466 proteins. B) Fold changes of 283 proteins differentially expressed in response to HT stimulus. C) Bar graph shows the–log q-value of enrichments of the top 5 pathways in the list of proteins differentially expressed after the HT stimulus. D) Fold changes of 379 proteins differentially expressed in response to the G stimulus. E) Bar graph shows the–log q-value of enrichments of the top 5 pathways in the list of proteins differentially expressed after the G stimulus.

While cells measure and respond to many environmental stimuli, we chose temperature and carbon source to test our hypothesis. Both stimuli are known to be important factors for survival and have wide-ranging effects on yeast metabolism [1]. We used growth with glucose at 30°C as the control, and high temperature and glycerol as the stimuli. The changing growth conditions were: glucose at 37°C (HT stimulus), glycerol at 30°C (G stimulus), and glycerol at 37°C concurrently (HT+G stimulus). To precisely measure the proteomic responses of the cell, we used isobaric tag for relative and absolute quantitation (iTRAQ) labeling followed by multi-dimensional protein identification technology (MudPIT)-based mass spectrometry to quantify the steady state proteomes under the four different growth conditions (S1 Fig) [40,41]. A total of 1064 proteins were quantitated in the control and the three test conditions. We filtered the data to focus only on the 466 proteins that were quantitated in all three independent replicates of all of the three test conditions (Fig 1A, S1 Table). Cross-correlation analysis of the filtered data showed high reproducibility among the replicates (S2 Fig). The proteomic changes in the cells grown with the concurrent stimuli were more similar to the changes induced by glycerol compared to high temperature (S2 Fig).

We defined the response to an environmental factor(s) as the log_2_-fold change in protein abundance/expression between the control and experimental conditions. For this study, we used “fold change” to denote the log_2_ fold change. We built linear regression models for each protein using fold changes to estimate the effect sizes of the stimuli. We used ANOVA for estimating statistical significances since we were comparing multiple stimuli. We interpreted the positive or negative sign of the effect size as either upregulation or downregulation, respectively. The Benjamini-Hochberg procedure was used to adjust the ANOVA *p-values* at 5% FDR [34]. A protein was assumed to be differentially expressed if the adjusted overall ANOVA *p-value* was less than 0.05. These proteins were further analyzed and classified into different environmental interaction classes using the direction of the change (upregulated or downregulated) and the *p-values* of the effect size estimates [34].

### Stimuli-specific expression patterns can be used to identify proteins important for responding to the stimuli.

We observed 283 proteins differentially expressed with high temperature, 379 proteins differentially expressed in response to glycerol, and 370 proteins were differentially expressed in concurrent high temperature and glycerol (Fig 1B and 1D, and S1 Table), while 41 proteins did not change in response to any of the stimuli. We selected GeneMANIA Cytoscape plugin for pathway analysis since it extends the input list of differentially expressed proteins by adding related proteins to enhance sensitivity and coverage [42,43]. It also allows using the complete proteome as the background. This helped to build a more complete picture of differentially regulated pathways. Pathway analysis of these two differentially expressed protein groups revealed the same top five pathways; none were specific to either stimulus (Fig 1C and 1E). All of the top five pathways were related to protein synthesis and translational control, suggesting that the regulation of protein synthesis is an important step in responding to environmental stimuli. Translation factors are some of the most abundant proteins in yeast and our proteomic assays are limited by the abundance of proteins in the cell. Although this could have confounded pathway analysis and led to the identification of translation associated pathways as being the most enriched, using only the differentially expressed proteins suggests that these pathways are, at the least, being differentially regulated. Furthermore, similar observations have also been made in previous studies [1,10,44]. It is noteworthy that the pathways expected to be important for responding to these stimuli, such as “protein folding” for growth at high temperature and “TCA cycle” for growth with glycerol were present farther down the list at numbers 39 and 53, respectively (S2 and S3 Tables) [45,46,47]. This mirrors a common problem in ‘omics’ studies that generate large lists of candidate genes, transcripts and proteins. The important responders are lost in a long list where a majority of differentially expressed genes or proteins is not directly responding to the stimulus. Therefore, choosing candidates for an in-depth mechanistic study becomes a challenge.

To address this problem, we devised a methodology using dominance in environmental interactions to identify proteins and pathways important for responding to a stimulus. We noticed proteomic expression patterns in which the response to one stimulus was dominant over the other. We speculated that a protein critical in responding to a stimulus will respond to that stimulus even when challenged by a competing stimulus. If this hypothesis is correct, such an environmental interaction could serve as a filter to select and identify proteins that respond specifically to the dominant environmental stimulus.

**Fig 2:**
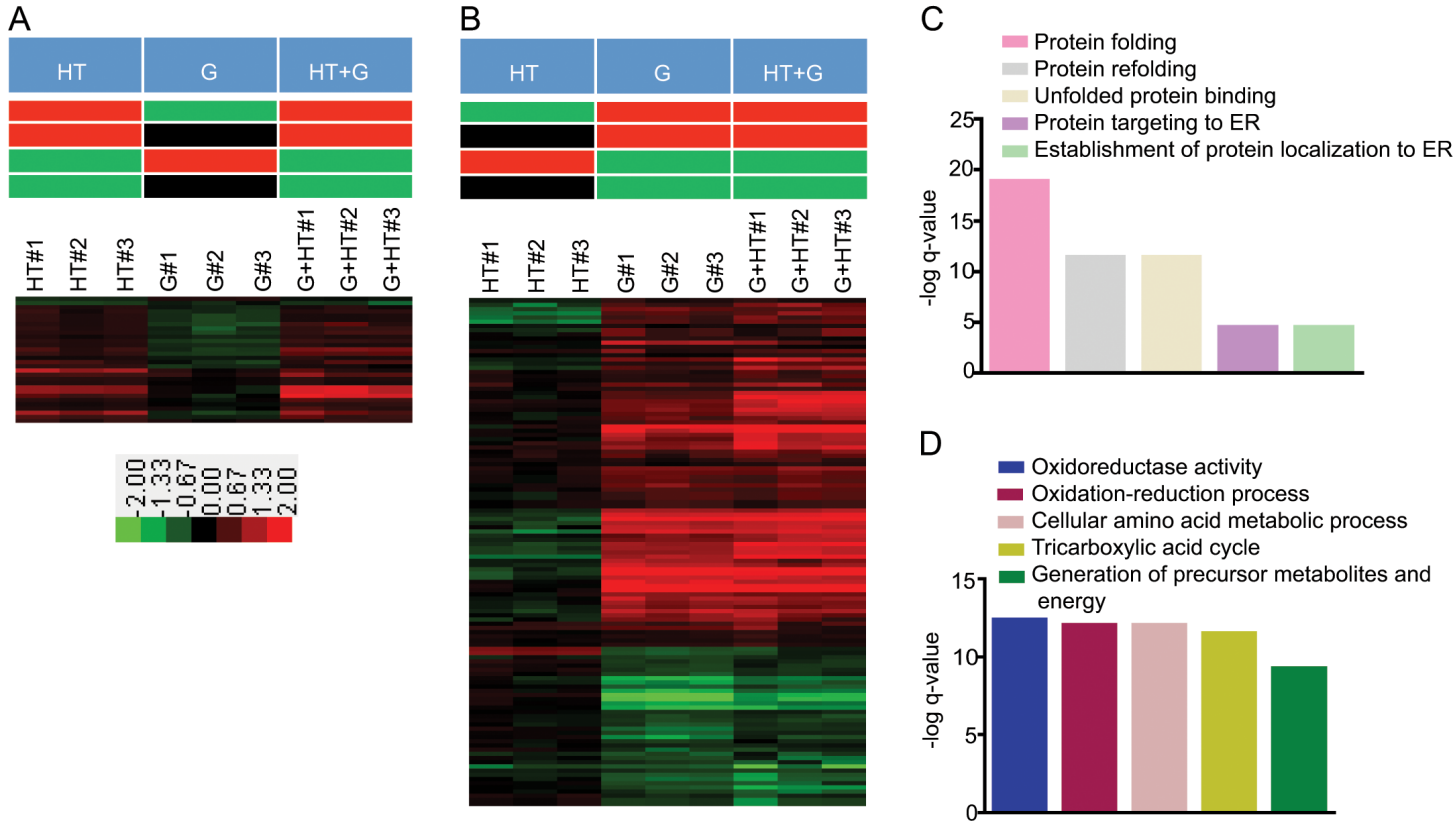
Dominance of an environmental stimulus used to identify proteins that are important for responding to the environmental stimulus. The color bar shows the range of fold changes. Pathway analysis was done using the *GeneMANIA* Cytoscape plugin [42]. Bar graphs were generated in *Graphpad Prism*. A) Proteins for which HT stimulus is dominant over G stimulus. The theoretical expression patterns are depicted in the top panel (Red, upregulation; green, downregulation; and black no statistically significant change in expression). The heatmap of fold changes in expression for 30 proteins for which HT stimulus is dominant over G stimulus is shown in bottom panel. B) Proteins for which G stimulus is dominant over HT stimulus. The theoretical expression patterns are depicted in the top panel. The heatmap of fold changes in expressions for 121 proteins for which G stimulus is dominant over HT stimulus is shown in bottom panel. C) Bar graph shows the–log q-value of enrichments of the top five pathways in the list of proteins for which HT stimulus is dominant over G stimulus. D) Bar graph shows the–log q-value of enrichments of the top five pathways in the list of proteins for which G stimulus is dominant over HT stimulus.

To test this hypothesis, we classified the list of 466 proteins responding to the concurrent glycerol and high temperature stimuli based upon their expression patterns. Two classes of dominant environmental interactions are possible. In one class, a stimulus reverses an expression change induced by the other stimulus (Fig 2A and 2B, top panels, rows 1 and 3). In the other class, a stimulus induces a change in expression, while the other stimulus has no effect on its own and does not change the response to the concurrent stimulus (Fig 2A and 2B top panels, rows 2 and 4). Each class is represented by two theoretical expression patterns for a total of four expression patterns for each stimulus (Fig 2A and 2B top panels).

For the environmental interactions in which the HT stimulus was dominant over the G stimulus, the *p-values* for all of the effect size estimates were less than 0.05. The changes for the HT and HT+G stimuli were in the same direction and differed from the G stimulus (Fig 2A, top panel, rows 1 and 3). Alternatively, the *p-values* for only the HT and HT+G stimuli effect size estimates were less than 0.05 and the directions of change for the HT and HT+G stimuli were the same (Fig 2A, top panel, rows 2 and 4). In all, we identified 30 proteins for which the response to the HT stimulus was dominant over the G stimulus (Fig 2A and S1 Table). We used pathway analysis to identify which protein classes were responding to the dominant stimulus. The group of proteins for which the HT stimulus was dominant included the heat shock response proteins HSP10, HSP60, SSA1, SSA2, and HSP150 (Fig 2A bottom panel, and S1 Table). Pathway analysis of these 30 proteins showed that the top five enriched pathways included protein folding, protein refolding, and unfolded protein binding (Fig 2C, S5 Table). These pathways are expected to be important for growth at higher temperatures [45,46,48,49].

For the environmental interaction in which the G stimulus is dominant, we saw a similar set of patterns as described above except the G stimulus dominates the HT stimulus (Fig 2B, top panel). There are 121 proteins for which the response to the G stimulus was dominant over the HT stimulus (Fig 2B, bottom panel and S1 Table). The group of proteins for which the G stimulus was dominant includes metabolic enzymes such as CDC19, ACO1, and LSC1. (Fig 2B, bottom panel, and S1 Table). Pathway analysis of these 121 proteins showed that the top five pathways included the oxidation-reduction process, the generation of precursor metabolites and energy, and the tricarboxylic acid cycle (Fig 2D, and S6 Table). All of these three pathways are expected to be important for respiratory growth [47,50,51]. Consistent with our hypothesis, pathway analysis of proteins that respond to a dominant environmental stimulus reveals a functional relationship to the response to the stimulus. High temperature has a dominant effect on proteins involved in protein folding, while glycerol has a dominant effect on proteins involved in respiratory metabolism. These results show the practical applications of using dominant environmental interactions to identify proteins that respond to specific stimuli and that are directly involved in the cell’s response to that stimulus.

### Analysis of expression patterns reveals that environmental interactions mirror gene interactions.

**Fig 3:**
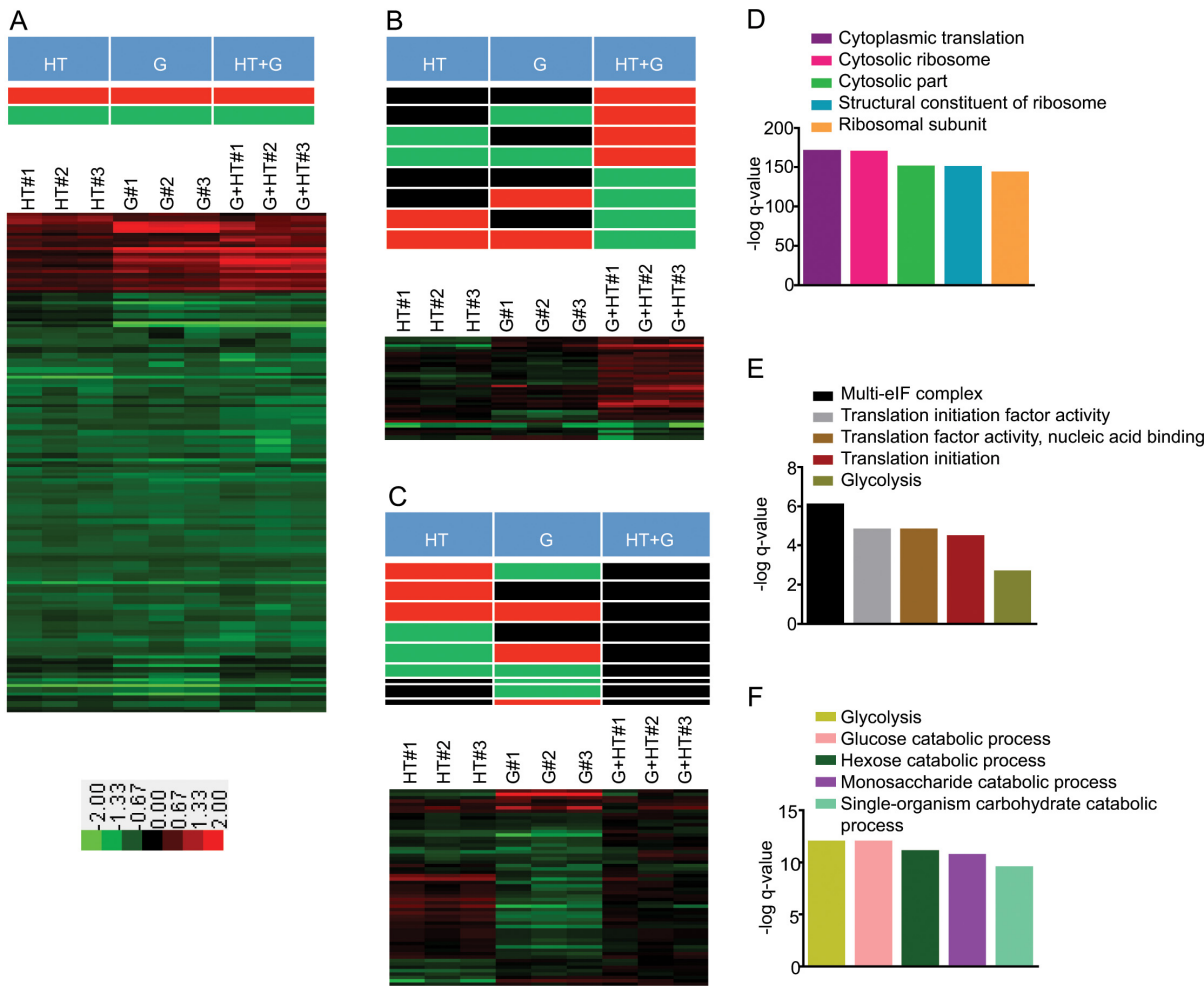
Proteins in different environmental interaction classes and the corresponding enriched pathways after concurrent G and HT stimuli. The color bar shows the range of fold changes. Pathway analysis was done using *GeneMANIA* Cytoscape plugin[42]. Bar graphs were generated in *Graphpad Prism*. A) Non-specific environmental response (NER) proteins to individual and concurrent HT and G environmental stimuli. The theoretical expression patterns are shown in the top panel. The fold changes of 175 NER proteins are shown as a heatmap. B) The theoretical expression patterns for discordant environmental interaction are shown in the top panel. The fold changes of 41 proteins are shown as a heatmap. C) The theoretical expression patterns for suppression environmental interaction are shown in the top panel. The fold changes of the 58 proteins affected by suppression are shown as a heatmap. D) Bar graph shows the–log q-value of enrichments for the top 5 pathways for the non-specific environmental response proteins. E) Bar graph shows the–log q-value of enrichments for the top 5 pathways in the list of proteins affected by discordant environmental interaction. F) Bar graph shows the–log q-value of enrichments for the top 5 pathways in the list of proteins affected by suppression environmental interaction.

In addition to the dominant interactions of concurrent environmental stimuli, we observed other classes of environmental interactions that mirror gene interactions. First, we observed a class of proteins whose abundance either increased or decreased in response to both the individual stimuli as well as the concurrent stimuli (Fig 3A). This is similar to gene pairs in which both the individual mutants as well as the double mutant have the same phenotype. We classified these proteins as non-specific environmental responders. This class is represented by two theoretical expression patterns: activated or repressed (Fig 3A, top panel and S1 Table). For these non-specific environmental response modules, the *p-values* for all the effect size estimates were less than 0.05 and the directions of change were the same (Fig 3A, top panel). We identified 175 proteins that correspond to these patterns, and pathway analysis revealed that they are largely involved in protein synthesis and translational control (Fig 3A, bottom panel and 3D, and S7 Table).

We also observed proteomic responses to concurrent environmental stimuli similar to gene interactions in which the two single mutants are wild-type or have one phenotype, while the double mutant has a different phenotype (Fig 3B). This class includes proteins whose expression was either decreased or unchanged after a single stimulus but was increased if both stimuli were applied concurrently. The class also includes proteins whose expression was either increased or unchanged after a single stimulus but was decreased by the concurrent stimuli. We classified this environmental interaction group as a discordant class. There are eight theoretical expression profiles in the discordant environmental interaction class (Fig 3B, top panel). For the discordant environmental interaction, the *p-value* for the HT+G concurrent stimuli effect size estimate was less than 0.05 and the directions of change for either the HT or G stimuli were not the same as HT+G. We identified 41 proteins that show discordance (Fig 3B, bottom panel and S1 Table). They are mainly involved in protein synthesis and metabolic pathways (Fig 3E, and S8 Table).

Finally, we observed suppression, in which a protein’s abundance changed in response to a single stimulus, yet the change was suppressed by the simultaneous application of the second stimulus (Fig 3C). This class is similar to gene interactions in which double mutants show the wild-type phenotype [52,53]. The suppression class is represented by eight theoretical expression patterns (Fig 3C, top panel). For suppression environmental interactions, the *p-value* for the HT+G effect size estimate was more than 0.05, and the *p-value* for at least one of HT and G stimuli effect size estimates was less than 0.05. We identified 58 proteins that are affected by suppression (Fig 3C, bottom panel and S1 Table). Pathway analysis revealed that metabolic pathways are most affected by suppression (Fig 3F and S9 Table).

### A large fraction of the proteome is affected by environmental epistasis

**Fig 4:**
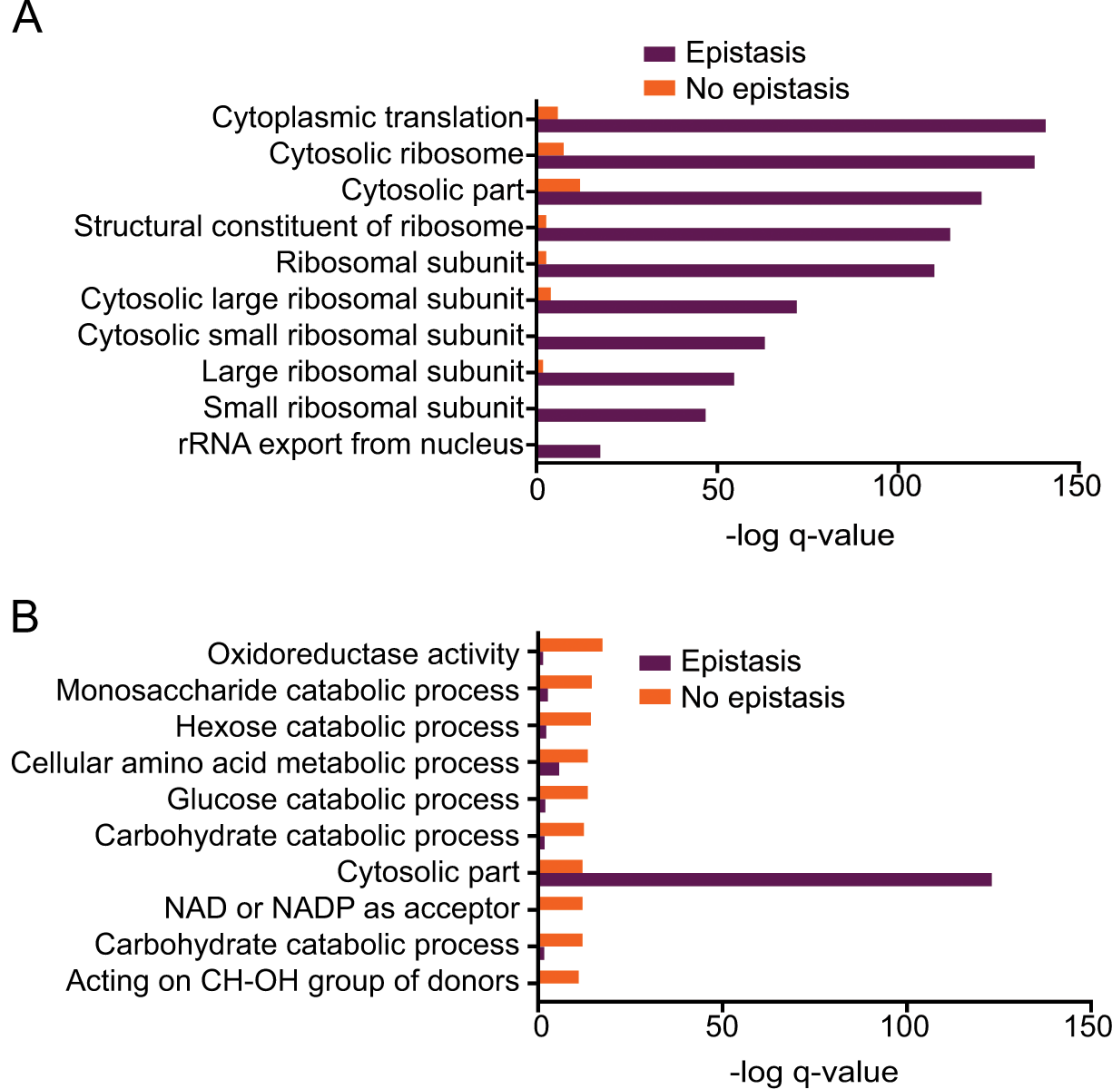
Environmental epistasis in the proteomic response to concurrent stimuli. Pathway analysis was done using the *GeneMANIA* Cytoscape plugin[42]. Bar graphs were generated in *Graphpad Prism*. A) Bar graph shows the–log q-value of enrichments of the top 10 pathways in the list of proteins affected by epistasis (purple) and their–log q-value in the list of proteins not affected by epistasis (orange). B) Bar graph shows the–log q-value of enrichments of the top 10 pathways in the list of proteins not affected by epistasis (orange) and their–log q-value in the list of proteins affected by epistasis (purple).

An important feature of genetic epistasis is that the modulating effects of multiple genes are not always independent of each other [20,21,22,54,55]. In many cases, non-independence is diagnostic of a functional relationship between genes [20,22,54]. Genetic epistasis is used to test if the effects of genetic elements are independent. Genetic epistasis occurs when the effects are not independent. We tested if the effects of these two individual environmental stimuli were independent of each other for individual proteins in the proteome. Similar to the mathematical approach to genetic epistasis, we measured the response of each protein and classified a response as influenced by environmental epistasis if the sum of the effects of the individual stimuli for a protein was not equal to the response to the concurrent stimuli (*t*-test, *p*-value ≤0.05) [20,21,22]. We used log_2_ fold change as the measure of the effect of a stimulus. From our list of 466 quantitated proteins, 240 proteins were affected by environmental epistasis (S1 Table). Pathway analysis of these proteins revealed that a majority of the enriched pathways are involved in protein synthesis and translational control (Fig 4A and S10 Table). The topmost enriched pathways included cytoplasmic translation, cytosolic ribosome, and structural constituent of ribosome (Fig 4A and S11 Table).

Pathway analysis of the 226 proteins not affected by environmental epistasis revealed a large number of metabolic pathways (Fig 4B and S1 and S10 Tables). It is interesting to note that the distribution of pathways affected by environmental epistasis is different from those that are unaffected. Protein synthesis and translational control seem to be disproportionately affected by environmental epistasis compared to other pathways. These pathways have previously been found to change in response to the changes in the growth rate [56,57]. If the effects of the two stimuli on the growth rate are not independent, it could explain the observed environmental epistasis. To test the independence in the effects of the two stimuli on the growth rate, we determined the doubling times under the same conditions. The change in the doubling times was used to measure the effect of a stimulus. Our data shows that the effects of high temperature and glycerol on the growth rate are additive and, therefore, independent of each other (S4 Fig). Further studies are required to elucidate the functional significance of the environmental epistasis.

A number of genetic epistasis subtypes have been defined based upon the mathematical models used to measure the expectation of a phenotype in double mutants [54,55,58,59,60]. Four most commonly used definitions are (1) additive, (2) multiplicative, (3) minimum, and (4) log [55,58]. Although we used only the additive definition for developing the concept of the environmental epistasis in this study, future studies can be performed to compare the results obtained using different definitions.

### Environmental interactions and epistasis regulate mRNA levels.

Although, we identified the environmental interactions using quantitative proteomic data, we speculated that this framework would be applicable to any quantifiable readout including transcriptomic and phenotypic traits. In pioneering studies using chemostat cultures of *S*. *cerevisiae*, Knijnenburg *et al*. measured the transcriptional response of yeast to multiple, concurrent environmental stimuli [13]. They found linear regression models of expression for the vast majority of genes required a combinatorial interaction term[13]. This suggests the change in transcription of most genes cannot be explained by simply adding the effects of the individual stimuli. Based on our proteomic results, we hypothesized that environmental epistasis plays a role in determining the state of the transcriptome as well.

**Fig 5:**
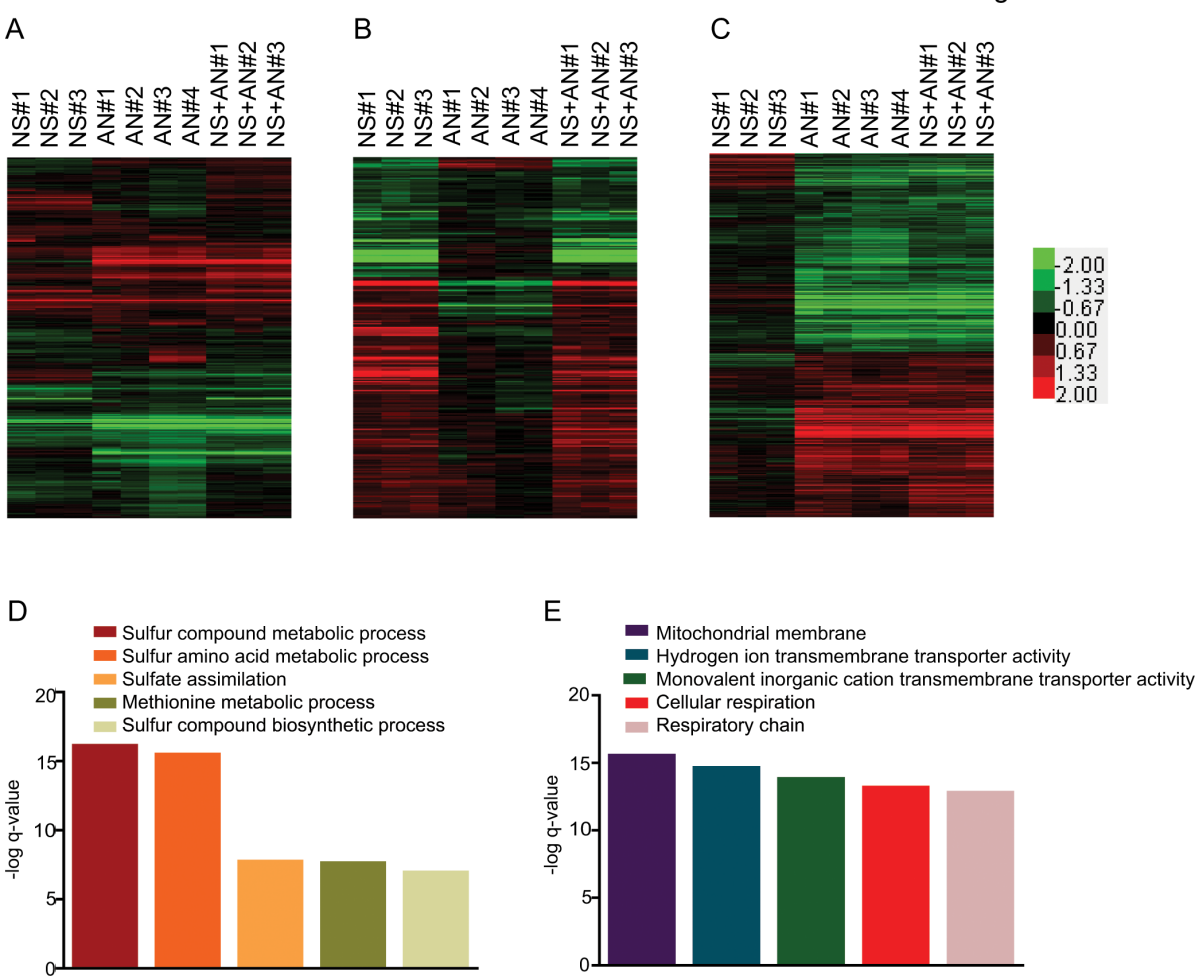
Environmental interactions affect transcriptomic profiles as well. Normalized expression data from *Knijnenburg et*. *al*. *2009* was used for the analyses. The transcriptomic data used in the study used haploid *S*. *cerevisiae* cells (CEN.PK113-7D MATa) grown in carbon limited chemostat cultures under 4 conditions – 1) ammonium sulfate as nitrogen source (n = 5), 2) methionine as nitrogen source, NS stimulus (n = 3), 3) Anaerobic condition, AN stimulus (n = 4), and 4) methionine as nitrogen source under anaerobic conditions NS+AN stimulus (n = 3) [13]. Fold changes were calculated from normalized expression data using average normalized expression data from the five replicates of growth with ammonium sulfate as the baseline. The color bar shows the range of fold changes. Pathway analysis was done using *GeneMANIA* Cytoscape plugin[42]. Bar graphs were generated in *Graphpad Prism*. A) A heatmap of fold changes of the complete transcriptomics dataset consisting of 6551 transcripts. B) A heatmap showing the fold changes for 281 transcripts for which NS stimulus is dominant. C) The–log q-value of enrichment for the top 5 pathways enriched in the list of transcripts for which NS stimulus is dominant. As anticipated, pathways expected to be involved in metabolization of methionine are enriched. D) A heatmap showing the fold changes for 938 transcripts for which AN stimulus is dominant. E) The–log q-value of enrichment for the top 5 pathways enriched in the list of transcripts for which AN stimulus is dominant. As anticipated, pathways expected to be involved in energy production are enriched.

To test if our environmental interaction and epistasis models are observed in the transcriptomic responses to concurrent stimuli, we analyzed Knijnenburg dataset which measured the transcriptomic responses of yeast cells growing in carbon limited chemostat cultures [13]. In the experiment, two concurrent stimuli were applied: (1) a change in nitrogen source from ammonium sulfate to methionine and (2) a change from aerobic to anaerobic growth (Fig 5A and S12 Table) [13]. The data showed 564 transcripts were affected by environmental epistasis, while 5987 transcripts were not affected (*p-value* ≤0.05) (S12 Table). In contrast to our proteomic analysis, pathway analysis of the transcripts affected by environmental epistasis revealed enrichment for pathways including microbody, peroxisome, and phytosteroid metabolic process (S13 Table). This could be because of the differences between the strains, stimuli, and culture conditions used in the transcriptomic and our proteomic studies. Similar to our proteomic analysis, we observed dominant environmental interactions in the expression of the transcripts (Fig 5B and 5C and S12 Table). Nitrogen source was dominant for 281 transcripts (Fig 5B and S12 Table). Pathway analysis of these transcripts identified pathways involved in methionine metabolism such as sulfur amino acid metabolic process, sulfur compound metabolic process and methionine metabolic process (Fig 5D and S14 Table). Similarly, anaerobic growth was dominant for 938 transcripts (Fig 5C and S12 Table). Pathway analysis of these differentially expressed transcripts showed enrichment of pathways involved in energy production such as cellular respiration, mitochondrial membrane and respiratory chain (Fig 5E and S15 Table). We also observed the same environmental interaction classes in their transcriptomic data as in our proteomic data, including non-specific environmental response, discordance, and suppression (S12 Table). These results strongly suggest that environmental interactions play a significant role in regulating the biochemical state of cells.

### Coexpression network analysis shows community structures are guided by environmental interaction and epistasis.

**Fig 6:**
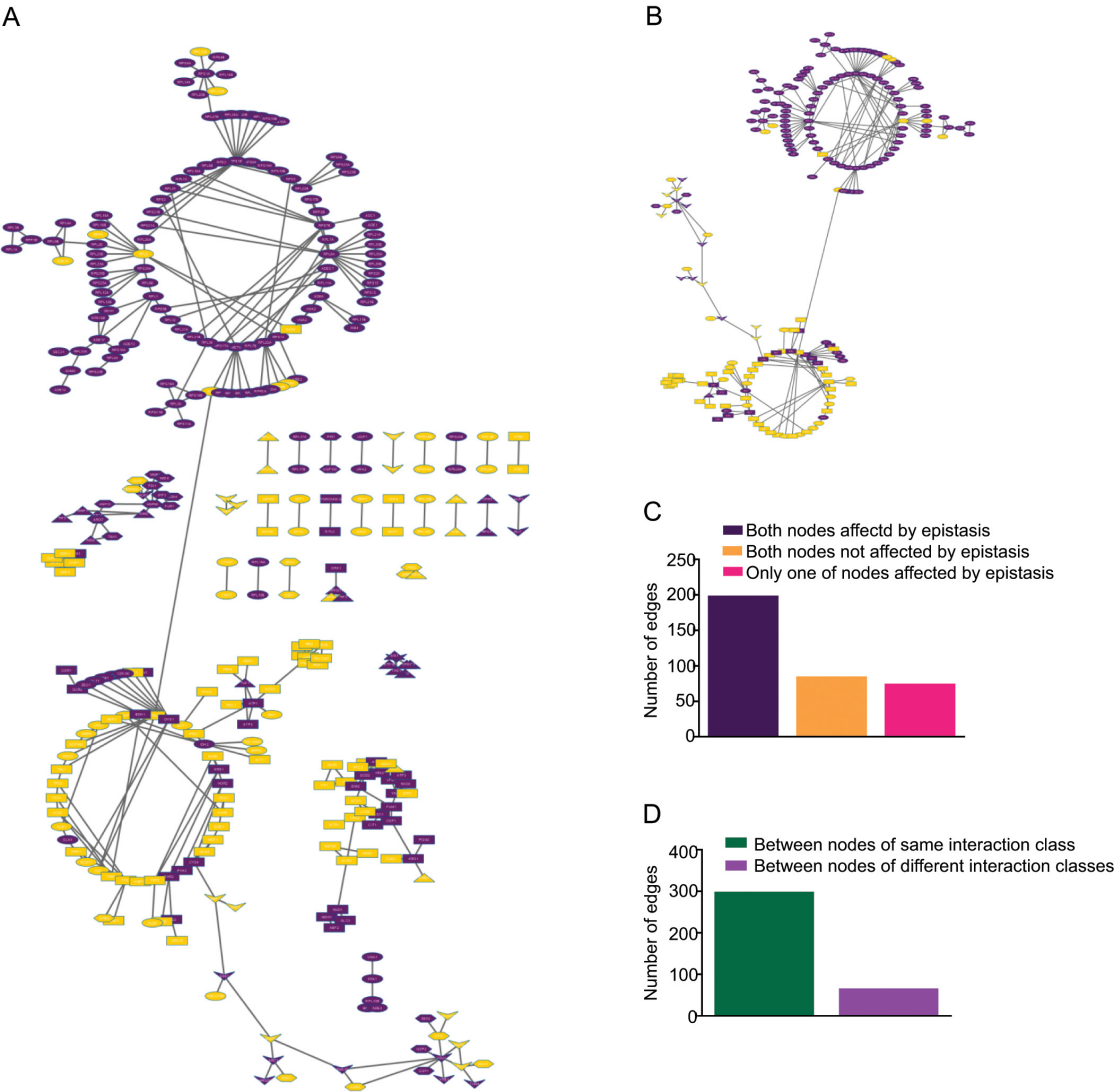
Coexpression network based on all the quantified proteins and all conditions (G stimulus, HT stimulus, and HT+G stimulus). Proteins are depicted as nodes. Nodes that are coexpressed are connected with an edge. The coexpression network was generated with *SPACE* algorithm using fold change [35]. Network visualization and analysis was done in Cytoscape 3.1.1 [39]. Bar graphs were generated in *Graphpad prism*. A) All nodes that have at least one edge. Nodes affected by environmental epistasis are highlighted in purple. The circular layout was used to generate the initial network graphics in Cytoscape 3.1.1. Far-flung communities of inter-connected nodes were manually brought together, while preserving the inner community structure, for better visualization. B) The largest community in the coexpression network. Most of the proteins affected by environmental epistasis are members of a subgraph (top circle) that predominantly contains other proteins that are also affected by environmental epistasis. A similar trend is observed with the proteins not affected by environmental epistasis (bottom circle). C) The numbers of three types edges: 1) both nodes are affected by environmental epistasis (199 edges), 2) neither of the nodes are affected by environmental epistasis (85 edges), and 3) only one of the nodes is affected by environmental epistasis (75 edges). Proteins affected by epistasis are predominantly connected to proteins that are also affected by epistasis. D) Number of edges that connect nodes to other nodes within the same environmental interaction classes (299) or between the classes (60). Co-regulatory connections between proteins are predominantly between those of the same class.

Coexpression networks link together proteins whose expression levels are regulated in the same way [61,62]. As a consequence, coexpression network analysis can be used to determine if the abundances of proteins affected by environmental epistasis are regulated differently than the proteins that are not affected by environmental epistasis. To explore the protein modules whose expression changes are correlated with each other, we built a coexpression network using the merged proteomic responses from both individual and concurrent stimuli using the Sparse PArtial Correlation Estimation approach (SPACE) (Fig 1A) [35]. An edge, representing coexpression, was introduced between two proteins if the correlation between them was above the average of the correlation matrix. To validate the network, we first tested the power law structure of the reconstructed network [35,63]. The reconstructed network followed the power law distribution. The power law parameter α was approximately 4, which is close to the empirically observed value of 3.45 [63]. Next, we repeatedly reconstructed the network by varying the tuning parameter around the default value and fitting the network to the power law distribution. We found that the reconstructed network follows the power law distribution and that the power law parameter was in the range of 3.75. We identified the sub-graph spanned by the top 1% of highly connected nodes. We found that the Jaccard similarity score of these highly connected nodes was 0.83 on the scale of 0 to 1. Therefore, these nodes were classified as hub nodes, which is one of the characteristic features of power law networks. There were 7 hub nodes based upon the above criterion. Next, we checked the significance of the identified hubs using the Wilcox Rank sum test and found that the hub community is statistically significant (*p*-value = 0.04) [64]. Finally, we compared the reconstructed network with BioGrid protein interaction data and found that approximately 30% of the edges are previously known interactions and that these interactions were found in every reconstructed network when we varied the tuning parameter to estimate the partial correlation matrix [65]. The final coexpression network consisted of 329 nodes with at least one neighbor and a total of 359 edges (Fig 6A).

The largest community within this network includes 205 nodes and 249 edges, with two clearly separate sub-graphs connected by a single node (Fig 6B). Interestingly, one sub-graph consists predominantly of proteins affected by environmental epistasis while the second sub-graph consists of proteins not affected by environmental epistasis. Within the global coexpression network, we observed that proteins affected by epistasis were more likely to be linked with each other than with proteins that are not affected by epistasis and vice versa (Fig 6A). There are 199 edges between two proteins affected by epistasis and 85 edges between two proteins not affected by epistasis. However, only 75 edges involved proteins of both types (Fig 6C). This structural organization of the coexpression network suggests that the responses of proteins affected by environmental epistasis are controlled by a different mechanism than the responses of those not affected by environmental epistasis.

Previous studies indicate that proteins linked in a coexpression network are likely to function in the same pathway [61]. We hypothesized that the grouping of proteins upon classification into environmental interaction classes might be driven by their functional associations. If true, we would expect to find more edges in the coexpression network between proteins within the environmental classes. Indeed, we found this result in this network. Our data show that 299 of the edges (83%) are between proteins in the same environmental interaction class, while only 60 are between proteins in different classes (Fig 6D).

## Discussion

Using the concepts of gene interactions and epistasis, we have developed a unifying conceptual framework to understand the cellular responses to complex environmental stimuli. Although, we have only explored the cases with complete dominance of a stimulus, it is possible that both the stimuli contribute to a change in expression. It is also possible that many stimuli contribute towards a change. We speculate that the tools and approaches developed for gene-gene interactions involving multiple genes can be applied in such cases [66]. In addition to linear regression modeling and ANOVA, we also tested our hypothesis using one sample and two sample *t-*tests of independence (data not shown). The results from both approaches were in good agreement.

The effect of mixtures of compounds has been actively studied in toxicology, especially in the context of environmental toxins [67,68,69,70,71,72,73,74,75,76,77,78]. These studies have led to the development of three complementary models to predict the combined effects of compounds in a mixture: (1) in the concentration addition model the total toxicity of a mixture is the sum of the individual toxicities of the component compounds, (2) in the independent action model the toxicities of the components of a mixture are independent of each other, and (3) in the simple interaction model the individual components, at the concentrations being tested, are not toxic, but are toxic when used together in a mixture. These models have been successful in predicting the total toxic effects of mixtures of compounds in many cases [67,69,70,71,72,74,78]. However, it is not immediately clear which one to apply in a specific case without model fitting [69].

Environmental interactions and epistasis can be extrapolated to explain the three models. For example, the concentration addition model can be the case of incomplete dominance where many stimuli affect the biological processes under investigation. This would happen if the compounds in the mixture affect similar biological pathways. If the actions of the compounds are antagonistic to each other, it may lead to either the dominance or the suppression interaction. If their actions are not antagonistic, the combined effect would be the sum of the individual effects which could be observed as the non-specific environmental response.

The independent action model explains the case where the compounds under investigation act upon different pathways [68,70,71,72,75,77]. This is similar to a gene interaction where two mutations have two unrelated phenotypes and both phenotypes persist in the double mutant. By applying the logic of environmental interaction to this model, we can deduce that the changes induced by a mixture that follows the independent action model would have elements specific to the component compounds of the mixture. Additionally, the changes important to a specific compound would persist in the combinatorial condition, which could be used to identify molecules and pathways that respond to the specific compound in the mixture.

The simple interaction model explains the cases where the compounds individually have little or no toxicity, but are toxic when applied together [73,75]. In terms of environmental interaction, this could be a case of the discordance interaction. The effects explained by this model could also be a special case of environmental epistasis, where the combined effect of compounds is more than the sum of their individual effects. It is worth noting that although we discuss only three of the mixture toxicity models, there are a number of other models that explain the toxicities of compounds in a mixture [67,68,69,70,71,72,73,74,75,76,77,78]. Environmental interactions and epistasis provides a conceptual framework unifying the different toxicity models. The interpretation of results can be made simpler using environmental interactions and epistasis.

Phenotypic plasticity provides the conceptual framework for studying the interaction between genotype and environment. Phenotypic plasticity is the ability of an organism to change its phenotype in response to changes in the environment [79]. It has been used to explain the ability of the same genotype to generate different phenotypes in different environments [79]. However, phenotypic plasticity considers the environment as a monolithic entity. It fails to separate the relative contributions of the different environment components, for example; physical components such as temperature and pressure, chemical components such as nutrients, and signaling molecules that activate different pathways. Applying environmental interactions and epistasis would help parse out the individual contributions of the stimuli towards the change in the phenotype.

Similar to genetic epistasis, our data show that the effects of individual environmental stimuli are not necessarily additive. Proteins affected by environmental epistasis are distributed throughout the genome and do not appear to be clustered at specific locations in the genome (S3 Fig). The prevalence of environmental epistasis in determining the changes in the proteome suggests that epistasis needs to be taken into account when building mathematical models of gene expression.

Consideration of environmental epistasis is especially important in light of the recent attempts to build quantitative linear regression models of gene expression in which the independent variables are the environmental stimuli and the dependent variable is gene expression [80]. Interestingly, in a linear regression modeling study of transcriptional regulation in rice under native conditions, the regression model was able to predict gene expression under native conditions even if the environmental parameters varied slightly from those used for building the model. However, the predictive power of the regression model was reduced under controlled laboratory conditions suggesting that there may have been unknown epistatic interactions in the native conditions absent in the controlled lab conditions [80].

Concurrently applied environmental stimuli behave similarly to genetic elements in the way they interact to regulate the biochemical states of the cells. The observation of environmental interactions and epistasis in determining the states of both the proteome and transcriptome in diverse experimental conditions suggests the prevalence of this phenomenon in nature. Essentially, environmental interaction in concert with phenotypic plasticity and gene interactions can be envisaged as a mathematical operator with three components that determines the changes in the biochemical state of the cell. The gene interaction component is derived from the effects of the genetic elements, while the environmental interaction component results from the effects of all the environmental stimuli. When the gene and environmental interactions are not independent of each other, phenotypic plasticity accounts for the deviations of the observed from the expected characteristic or trait. Most studies so far have treated phenotypic plasticity, gene interactions, and environmental interactions separately due to a lack of a common unifying framework [20,22,53,54,55,68,69,70,72,75,79,81,82,83,84,85]. Our data suggest that as an abstraction, environmental stimuli can be treated as genes to build a conceptual framework that combines the effects of genes and stimuli. Environmental interactions and epistasis play a critical role in cellular homeostasis as seen in this study’s patterns of change in the proteome and the transcriptome.

Our data also suggest that a protein or a transcript is more likely to be critical for responding to a dominant environmental stimulus than to a recessive one. This could lead to more efficient experiment designs for identifying factors directly affected by an environmental stimulus. For example, experiments could be designed in which an unrelated stimulus B is applied concurrently with the stimulus of interest A. The proteins or transcripts, for which the effect of A is dominant, would be more likely to be directly affected by stimulus A. We speculate that the same approach may be extended to genetic perturbations. In this case, an environmental stimulus could be applied in conjunction with the genetic perturbation. As with two concurrent environmental stimuli, a transcript or a protein for which the genetic perturbation is dominant may be more likely to be directly affected by it. Therefore, using dominance, environmental interactions can also be used to devise studies to identify agents, such as regulatory RNAs, proteins, or small molecules which are critical for driving a range of biological processes in health and disease including drug interactions, adaptation in tumor microenvironment and immune responses.

## Supporting Information

S1 Fig Experimental design workflow used in this study. Two environmental stimuli used were high temperature and glycerol as the carbon source. Diploid *S*. *cerevisiae* cells (BY4743) were grown in rich media under 4 conditions: 1) glucose at 30°C (used as control), 2) glycerol at 30°C (G stimulus), 3) glucose at 37°C (HT stimulus), and 4) glycerol at 37°C (HT+G stimuli). Three biological replicates were performed for each condition.
(EPS)

S2 Fig Correlation matrix heatmap (red is high). Correlation matrix was generated in *R*. There is a high correlation among replicates showing reproducibility across experimental replicates.
(EPS)

S3 Fig Visualization of *S*. *cerevisiae* genomic locations of the proteins quantitated with fold changes represented as a heatmap using Circos plot (Red: Up, Green: Down, Black: No change)[32]. Outermost circle- chromosomes, Second circle-fold changes of proteins with HT stimulus, Third circle-fold changes of proteins with G stimulus, Fourth circle-fold changes of proteins with HT+G stimuli, innermost circle-whether affected by epistasis or not (Purple: Affected by environmental epistasis, Orange: Not affected by environmental epistasis).
(EPS)

S4 Fig The effect of high temperature and glycerol on yeast doubling times. Doubling times were calculated for growth in control (n = 25), high temperature (n = 25), glycerol (n = 25), and concurrent high temperature and glycerol (n = 24). The difference in doubling times from the control was used to measure the effect of the stimuli and is plotted on Y-axis. HT leads to a decrease of -11 minutes (sd = 6), G leads to an increase of 137 minutes (sd = 14), and HT+G leads to an increase of 142. minutes (sd = 22). The expected effect of HT+G was calculated by summing the observed effects of HT and G (Sum HT+G, increase of 127 minutes with sd of 16). The difference in the means for HT+G and Sum HT+G was not statistically significant (*p-value* = 0.1034, two-tailed *t*-test of independence with Bonferroni correction for 11 comparisons)
(EPS)

S1 Table Complete data matrix of proteins. (TXT)

S2 Table GeneMANIA pathway analysis output for HT stimulus. (XLSX)

S3 Table GeneMANIA pathway analysis output for G stimulus. (XLSX)

S4 Table GeneMANIA pathway analysis output for HT+G stimulus. (XLSX)

S5 Table GeneMANIA pathway analysis output for HT stimulus dominance. (XLSX)

S6 Table GeneMANIA pathway analysis output for G stimulus dominance. (XLSX)

S7 Table GeneMANIA pathway analysis output for non-specific environmental reponse in protein expression. (XLSX)

S8 Table GeneMANIA pathway analysis output for discordance in protein expression. (XLSX)

S9 Table GeneMANIA pathway analysis output for suppression in protein expression. (XLSX)

S10 Table GeneMANIA pathway analysis output for environmental epistasis in protein expression. (XLSX)

S11 Table GeneMANIA pathway analysis output for no environmental epistasis in protein expression. (XLSX)

S12 Table Complete data matrix of transcripts (TXT)

S13 Table GeneMANIA pathway analysis output for environmental epistasis in transcript expression. (XLSX)

S14 Table GeneMANIA pathway analysis output for dominance of NS (XLSX)

S15 Table GeneMANIA pathway analysis output for dominance of AN (XLSX)

S16 Table Doubling times under the 8 growth conditions. (XLSX)

## References

[1] Gasch AP, Spellman PT, Kao CM, Carmel-Harel O, Eisen MB, et al (2000) Genomic expression programs in the response of yeast cells to environmental changes. Mol Biol Cell 11: 4241–4257. 11102521 10.1091/mbc.11.12.4241 PMC15070

[2] Gerner C, Vejda S, Gelbmann D, Bayer E, Gotzmann J, et al (2002) Concomitant determination of absolute values of cellular protein amounts, synthesis rates, and turnover rates by quantitative proteome profiling. Mol Cell Proteomics 1: 528–537. 12239281 10.1074/mcp.m200026-mcp200

[3] Pratt JM, Petty J, Riba-Garcia I, Robertson DH, Gaskell SJ, et al (2002) Dynamics of protein turnover, a missing dimension in proteomics. Mol Cell Proteomics 1: 579–591. 12376573 10.1074/mcp.m200046-mcp200

[4] Soufi B, Kelstrup CD, Stoehr G, Frohlich F, Walther TC, et al (2009) Global analysis of the yeast osmotic stress response by quantitative proteomics. Mol Biosyst 5: 1337–1346. 10.1039/b902256b 19823750

[5] Yan SP, Zhang QY, Tang ZC, Su WA, Sun WN (2006) Comparative proteomic analysis provides new insights into chilling stress responses in rice. Mol Cell Proteomics 5: 484–496. 16316980 10.1074/mcp.M500251-MCP200

[6] Hazlehurst LA, Landowski TH, Dalton WS (2003) Role of the tumor microenvironment in mediating de novo resistance to drugs and physiological mediators of cell death. Oncogene 22: 7396–7402. 14576847 10.1038/sj.onc.1206943

[7] Trédan O, Galmarini CM, Patel K, Tannock IF (2007) Drug Resistance and the Solid Tumor Microenvironment. Journal of the National Cancer Institute 99: 1441–1454. 17895480 10.1093/jnci/djm135

[8] Vaupel P, Kallinowski F, Okunieff P (1989) Blood Flow, Oxygen and Nutrient Supply, and Metabolic Microenvironment of Human Tumors: A Review. Cancer Research 49: 6449–6465. 2684393

[9] Whiteside TL (2008) The tumor microenvironment and its role in promoting tumor growth. Oncogene 27: 5904–5912. 10.1038/onc.2008.271 18836471 PMC3689267

[10] Brauer MJ, Huttenhower C, Airoldi EM, Rosenstein R, Matese JC, et al (2008) Coordination of growth rate, cell cycle, stress response, and metabolic activity in yeast. Mol Biol Cell 19: 352–367. 17959824 10.1091/mbc.E07-08-0779 PMC2174172

[11] De Nicola R, Hazelwood LA, De Hulster EAF, Walsh MC, Knijnenburg TA, et al (2007) Physiological and Transcriptional Responses of Saccharomyces cerevisiae to Zinc Limitation in Chemostat Cultures. Appl Environ Microbiol 73: 7680–7692. 17933919 10.1128/AEM.01445-07 PMC2168061

[12] Kanani H, Dutta B, Klapa MI (2010) Individual vs. combinatorial effect of elevated CO2 conditions and salinity stress on Arabidopsis thaliana liquid cultures: comparing the early molecular response using time-series transcriptomic and metabolomic analyses. BMC Syst Biol 4: 177 10.1186/1752-0509-4-177 21190570 PMC3027597

[13] Knijnenburg TA, Daran JM, van den Broek MA, Daran-Lapujade PA, de Winde JH, et al (2009) Combinatorial effects of environmental parameters on transcriptional regulation in Saccharomyces cerevisiae: a quantitative analysis of a compendium of chemostat-based transcriptome data. BMC Genomics 10: 53 10.1186/1471-2164-10-53 19173729 PMC2640415

[14] Knijnenburg TA, de Winde JH, Daran JM, Daran-Lapujade P, Pronk JT, et al (2007) Exploiting combinatorial cultivation conditions to infer transcriptional regulation. BMC Genomics 8: 25 17241460 10.1186/1471-2164-8-25 PMC1797021

[15] Murray JI, Whitfield ML, Trinklein ND, Myers RM, Brown PO, et al (2004) Diverse and specific gene expression responses to stresses in cultured human cells. Mol Biol Cell 15: 2361–2374. 15004229 10.1091/mbc.E03-11-0799 PMC404029

[16] Tai SL, Boer VM, Daran-Lapujade P, Walsh MC, de Winde JH, et al (2005) Two-dimensional Transcriptome Analysis in Chemostat Cultures: Combinatorial effects of oxygen availability and macronutrient limitation in *Saccharomyces cerevisiae* . Journal of Biological Chemistry 280: 437–447. 15496405 10.1074/jbc.M410573200

[17] Vaga S, Bernardo-Faura M, Cokelaer T, Maiolica A, Barnes CA, et al (2014) Phosphoproteomic analyses reveal novel cross-modulation mechanisms between two signaling pathways in yeast. Mol Syst Biol 10: 767 10.15252/msb.20145112 25492886 PMC4300490

[18] Pierce B (2005) Genetics: A conceptual approach: W. H. Freeman and Company.

[19] Bateson W (1909) Mendel's Principles of Heredity, by Bateson W.: Cambridge [Eng.] University Press,1909.

[20] Cordell HJ (2002) Epistasis: what it means, what it doesn't mean, and statistical methods to detect it in humans. Hum Mol Genet 11: 2463–2468. 12351582 10.1093/hmg/11.20.2463

[21] Fisher RA, Sir. (1958) The genetical theory of natural selection New York: Dover Publications.

[22] Phillips PC (2008) Epistasis—the essential role of gene interactions in the structure and evolution of genetic systems. Nat Rev Genet 9: 855–867. 10.1038/nrg2452 18852697 PMC2689140

[23] Baker Brachmann C, Davies A, Cost GJ, Caputo E, Li J, et al (1998) Designer deletion strains derived from Saccharomyces cerevisiae S288C: A useful set of strains and plasmids for PCR-mediated gene disruption and other applications. Yeast 14: 115–132. 9483801 10.1002/(SICI)1097-0061(19980130)14:2<115::AID-YEA204>3.0.CO;2-2

[24] Amberg DC, Burke DJ, Strathern JN (2005) Methods in Yeast Genetics: A Cold Spring Harbor Laboratory Course Manual, 2005 Edition Long Island, New York.: Cold Spring Harbor Laboratory Press.

[25] Dunn OJ (1961) Multiple Comparisons among Means. J Am Stat Assoc 56: 52–64.

[26] Browne CM, Samir P, Fites JS, Villarreal SA, Link AJ (2013) The Yeast Eukaryotic Translation Initiation Factor 2B Translation Initiation Complex Interacts with the Fatty Acid Synthesis Enzyme YBR159W and Endoplasmic Reticulum Membranes. Mol Cell Biol 33: 1041–1056. 10.1128/MCB.00811-12 23263984 PMC3623073

[27] Hoek KL, Samir P, Howard LM, Niu X, Prasad N, et al (2015) A cell-based systems biology assessment of human blood to monitor immune responses after influenza vaccination. PLoS One 10: e0118528 10.1371/journal.pone.0118528 25706537 PMC4338067

[28] Eng JK, Fischer B, Grossmann J, MacCoss MJ (2008) A Fast SEQUEST Cross Correlation Algorithm. Journal of Proteome Research 7: 4598–4602. 10.1021/pr800420s 18774840

[29] Eng JK, McCormack AL, Yates JR (1994) An Approach to Correlate Tandem Mass-Spectral Data of Peptides With Amino-Acid-Sequences in a Protein Database. Journal of the American Society for Mass Spectrometry 5: 976–989. 10.1016/1044-0305(94)80016-2 24226387

[30] Eisen MB, Spellman PT, Brown PO, Botstein D (1998) Cluster Analysis and Display of Genome-Wide Expression Patterns. Proceedings of the National Academy of Sciences 95: 14863–14868. 10.1073/pnas.95.25.14863 PMC24541 9843981

[31] Saldanha AJ (2004) Java Treeview—extensible visualization of microarray data. Bioinformatics 20: 3246–3248. 15180930 10.1093/bioinformatics/bth349

[32] Krzywinski M, Schein J, Birol I, Connors J, Gascoyne R, et al (2009) Circos: an information aesthetic for comparative genomics. Genome Res 19: 1639–1645. 10.1101/gr.092759.109 19541911 PMC2752132

[33] Vizcaino JA, Deutsch EW, Wang R, Csordas A, Reisinger F, et al (2014) ProteomeXchange provides globally coordinated proteomics data submission and dissemination. Nat Biotechnol 32: 223–226. 10.1038/nbt.2839 24727771 PMC3986813

[34] Benjamini Y, Hochberg Y (1995) Controlling the False Discovery Rate: A Practical and Powerful Approach to Multiple Testing. Journal of the Royal Statistical Society Series B (Methodological) 57: 289–300.

[35] Peng J, Wang P, Zhou N, Zhu J (2009) Partial Correlation Estimation by Joint Sparse Regression Models. J Am Stat Assoc 104: 735–746. 19881892 10.1198/jasa.2009.0126 PMC2770199

[36] Dieterle F, Ross A, Schlotterbeck G, Senn H (2006) Probabilistic quotient normalization as robust method to account for dilution of complex biological mixtures. Application in 1H NMR metabonomics. Anal Chem 78: 4281–4290. 16808434 10.1021/ac051632c

[37] Durbin BP, Hardin JS, Hawkins DM, Rocke DM (2002) A variance-stabilizing transformation for gene-expression microarray data. Bioinformatics 18 Suppl 1: S105–110. 12169537 10.1093/bioinformatics/18.suppl_1.s105

[38] van den Berg RA, Hoefsloot HC, Westerhuis JA, Smilde AK, van der Werf MJ (2006) Centering, scaling, and transformations: improving the biological information content of metabolomics data. BMC Genomics 7: 142 16762068 10.1186/1471-2164-7-142 PMC1534033

[39] Shannon P, Markiel A, Ozier O, Baliga NS, Wang JT, et al (2003) Cytoscape: a software environment for integrated models of biomolecular interaction networks. Genome Res 13: 2498–2504. 14597658 10.1101/gr.1239303 PMC403769

[40] Link AJ, Eng J, Schieltz DM, Carmack E, Mize GJ, et al (1999) Direct analysis of protein complexes using mass spectrometry. Nat Biotechnol 17: 676–682. 10404161 10.1038/10890

[41] Ross PL, Huang YLN, Marchese JN, Williamson B, Parker K, et al (2004) Multiplexed protein quantitation in Saccharomyces cerevisiae using amine-reactive isobaric tagging reagents. Molecular & Cellular Proteomics 3: 1154–1169. 15385600 10.1074/mcp.M400129-MCP200

[42] Montojo J, Zuberi K, Rodriguez H, Kazi F, Wright G, et al (2010) GeneMANIA Cytoscape plugin: fast gene function predictions on the desktop. Bioinformatics 26: 2927–2928. 10.1093/bioinformatics/btq562 20926419 PMC2971582

[43] Mostafavi S, Ray D, Warde-Farley D, Grouios C, Morris Q (2008) GeneMANIA: a real-time multiple association network integration algorithm for predicting gene function. Genome Biol 9 Suppl 1: S4 10.1186/gb-2008-9-s1-s4 18613948 PMC2447538

[44] Roberts G, Hudson A (2006) Transcriptome profiling of Saccharomyces cerevisiae during a transition from fermentative to glycerol-based respiratory growth reveals extensive metabolic and structural remodeling. Molecular Genetics and Genomics 276: 170–186. 16741729 10.1007/s00438-006-0133-9

[45] Richter K, Haslbeck M, Buchner J (2010) The Heat Shock Response: Life on the Verge of Death. Molecular Cell 40: 253–266. 10.1016/j.molcel.2010.10.006 20965420

[46] Riezman H (2004) Why Do Cells Require Heat Shock Proteins to Survive Heat Stress? Cell Cycle 3: 60–62. 14657667

[47] Schüller H-J (2003) Transcriptional control of nonfermentative metabolism in the yeast Saccharomyces cerevisiae. Current Genetics 43: 139–160. 12715202 10.1007/s00294-003-0381-8

[48] Åkerfelt M, Morimoto RI, Sistonen L (2010) Heat Shock Factors: Integrators of Cell Stress, Development and Lifespan. Nat Rev Mol Cell Biol 11: 545–555. 10.1038/nrm2938 20628411 PMC3402356

[49] de Nadal E, Ammerer G, Posas F (2011) Controlling gene expression in response to stress. Nat Rev Genet 12: 833–845. 10.1038/nrg3055 22048664

[50] Brisson D, Vohl M-C, St-Pierre J, Hudson TJ, Gaudet D (2001) Glycerol: a neglected variable in metabolic processes? BioEssays 23: 534–542. 11385633 10.1002/bies.1073

[51] Nevoigt E, Stahl U (1997) Osmoregulation and glycerol metabolism in the yeast Saccharomyces cerevisiae. Fems Microbiology Reviews 21: 231–241. 9451815 10.1111/j.1574-6976.1997.tb00352.x

[52] Dixon SJ, Costanzo M, Baryshnikova A, Andrews B, Boone C (2009) Systematic mapping of genetic interaction networks. Annu Rev Genet 43: 601–625. 10.1146/annurev.genet.39.073003.114751 19712041

[53] St Johnston D (2002) The art and design of genetic screens: Drosophila melanogaster. Nat Rev Genet 3: 176–188. 11972155 10.1038/nrg751

[54] de Visser JA, Cooper TF, Elena SF (2011) The causes of epistasis. Proc Biol Sci 278: 3617–3624. 10.1098/rspb.2011.1537 21976687 PMC3203509

[55] Mani R, St Onge RP, Hartman JLt, Giaever G, Roth FP (2008) Defining genetic interaction. Proc Natl Acad Sci U S A 105: 3461–3466. 10.1073/pnas.0712255105 18305163 PMC2265146

[56] Regenberg B, Grotkjaer T, Winther O, Fausboll A, Akesson M, et al (2006) Growth-rate regulated genes have profound impact on interpretation of transcriptome profiling in Saccharomyces cerevisiae. Genome Biol 7: R107 17105650 10.1186/gb-2006-7-11-r107 PMC1794586

[57] Slavov N, Botstein D (2011) Coupling among growth rate response, metabolic cycle, and cell division cycle in yeast. Mol Biol Cell 22: 1997–2009. 10.1091/mbc.E11-02-0132 21525243 PMC3113766

[58] Gao H, Granka JM, Feldman MW (2010) On the classification of epistatic interactions. Genetics 184: 827–837. 10.1534/genetics.109.111120 20026678 PMC2845349

[59] Hallgrimsdottir IB, Yuster DS (2008) A complete classification of epistatic two-locus models. BMC Genet 9: 17 10.1186/1471-2156-9-17 18284682 PMC2289835

[60] Li W, Reich J (2000) A complete enumeration and classification of two-locus disease models. Hum Hered 50: 334–349. 10899752 10.1159/000022939

[61] Stuart JM, Segal E, Koller D, Kim SK (2003) A gene-coexpression network for global discovery of conserved genetic modules. Science 302: 249–255. 12934013 10.1126/science.1087447

[62] Zhang B, Horvath S (2005) A general framework for weighted gene co-expression network analysis. Stat Appl Genet Mol Biol 4: Article17. 10.2202/1544-6115.1128 16646834

[63] Clauset A, Shalizi C, Newman M (2009) Power-Law Distributions in Empirical Data. SIAM Review 51: 661–703.

[64] Kolaczyk ED, Csárdi G (2014) Statistical Analysis of Network Data with R: Springer.

[65] Stark C, Breitkreutz BJ, Reguly T, Boucher L, Breitkreutz A, et al (2006) BioGRID: a general repository for interaction datasets. Nucleic Acids Res 34: D535–539. 16381927 10.1093/nar/gkj109 PMC1347471

[66] Cordell HJ (2009) Detecting gene-gene interactions that underlie human diseases. Nature Reviews Genetics 10: 392–404. 10.1038/nrg2579 19434077 PMC2872761

[67] Hermens J, Leeuwangh P, Musch A (1985) Joint toxicity of mixtures of groups of organic aquatic pollutants to the guppy (Poecilia reticulata). Ecotoxicology and Environmental Safety 9: 321–326. 4006831 10.1016/0147-6513(85)90049-1

[68] Altenburger R, Backhaus T, Boedeker W, Faust M, Scholze M (2013) Simplifying complexity: Mixture toxicity assessment in the last 20 years. Environ Toxicol Chem 32: 1685–1687. 10.1002/etc.2294 23843317

[69] Belden JB, Gilliom RJ, Lydy MJ (2007) How well can we predict the toxicity of pesticide mixtures to aquatic life? Integr Environ Assess Manag 3: 364–372. 17695109

[70] Altenburger R, Nendza M, Schuurmann G (2003) Mixture toxicity and its modeling by quantitative structure-activity relationships. Environ Toxicol Chem 22: 1900–1915. 12924589 10.1897/01-386

[71] Altenburger R, Scholz S, Schmitt-Jansen M, Busch W, Escher BI (2012) Mixture toxicity revisited from a toxicogenomic perspective. Environ Sci Technol 46: 2508–2522. 10.1021/es2038036 22283441

[72] Altenburger R, Walter H, Grote M (2004) What contributes to the combined effect of a complex mixture? Environ Sci Technol 38: 6353–6362. 15597892 10.1021/es049528k

[73] Berenbaum MC (1989) What is synergy? Pharmacol Rev 41: 93–141. 2692037

[74] Deneer JW (2000) Toxicity of mixtures of pesticides in aquatic systems. Pest Management Science 56: 516–520.

[75] Greco WR, Bravo G, Parsons JC (1995) The search for synergy: a critical review from a response surface perspective. Pharmacol Rev 47: 331–385. 7568331

[76] Lee JH, Landrum PF (2006) Development of a multi-component Damage Assessment Model (MDAM) for time-dependent mixture toxicity with toxicokinetic interactions. Environ Sci Technol 40: 1341–1349. 16572795 10.1021/es051120f

[77] Schoen ED (1996) Statistical designs in combination toxicology: a matter of choice. Food Chem Toxicol 34: 1059–1065. 9119316 10.1016/s0278-6915(97)00075-6

[78] Faust M, Altenburger R, Backhaus T, Blanck H, Boedeker W, et al (2001) Predicting the joint algal toxicity of multi-component s-triazine mixtures at low-effect concentrations of individual toxicants. Aquat Toxicol 56: 13–32. 11690628 10.1016/s0166-445x(01)00187-4

[79] Scheiner SM (1993) Genetics and Evolution of Phenotypic Plasticity. Annual Review of Ecology and Systematics 24: 35–68.

[80] Nagano AJ, Sato Y, Mihara M, Antonio BA, Motoyama R, et al (2012) Deciphering and prediction of transcriptome dynamics under fluctuating field conditions. Cell 151: 1358–1369. 10.1016/j.cell.2012.10.048 23217716

[81] Via S, Lande R (1985) Genotype-Environment Interaction and the Evolution of Phenotypic Plasticity. Evolution 39: 505–522. 28561964 10.1111/j.1558-5646.1985.tb00391.x

[82] Gerard JF, Vancassel M, Laffort B (1993) Spread of phenotypic plasticity or genetic assimilation: the possible role of genetic constraints. J Theor Biol 164: 341–349. 8246523 10.1006/jtbi.1993.1158

[83] Schlichting CD, Pigliucci M (1993) Control of phenotypic plasticity via regulatory genes. Am Nat 142: 366–370. 10.1086/285543 19425982

[84] Wilson M, Lindow SE (1993) Effect of phenotypic plasticity on epiphytic survival and colonization by Pseudomonas syringae. Appl Environ Microbiol 59: 410–416. 8434910 10.1128/aem.59.2.410-416.1993 PMC202120

[85] Tonsor SJ, Elnaccash TW, Scheiner SM (2013) Developmental instability is genetically correlated with phenotypic plasticity, constraining heritability, and fitness. Evolution 67: 2923–2935. 10.1111/evo.12175 24094343

